# Diagnosis and management of root resorption by erupting canines using cone-beam computed tomography and fixed palatal appliance: a case report

**DOI:** 10.1186/1752-1947-4-399

**Published:** 2010-12-09

**Authors:** Bodore K Albaker, Ricky WK Wong

**Affiliations:** 1 Orthodontics, Faculty of Dentistry, the University of Hong Kong, Hong Kong SAR, PR China

## Abstract

**Introduction:**

Resorption of the root of the maxillary incisors during ectopic eruption of the maxillary canines is not an uncommon phenomenon, and must be considered in all patients with seriously diverging eruption of the maxillary canines.

**Case presentation:**

We report on the diagnosis and treatment of a 10-year-old Chinese boy with severe crowding and risk of root resorptions caused by impacted canines in the upper arch and reverse overjet. With the aid of cone-beam computed tomography, the upper right canine crown of our patient was positioned in close proximity to the right lateral incisor while the left canine crown was hitting the root apex of the left lateral incisor. To avoid any progress of root resorption, use of an upper fixed palatal appliance with torquing spring to move the root of lateral incisors away from the canines, plus extraction of upper primary first molars, was selected as an interceptive treatment.

**Conclusions:**

Careful planning is crucial to avoid any complication through orthodontic treatment and to reduce the treatment time and cost.

## Introduction

Resorption of the root of the maxillary incisors during ectopic eruption of the maxillary canines is not an uncommon phenomenon, and must be considered in all patients with seriously diverging eruption of the maxillary canines. The root of the lateral incisor is the most commonly affected by resorption, although the central incisors can be affected [1-3]. Incisor resorption has been reported to occur more frequently in women, with the female to male ratio being reported as 2:1 [3], 4:1 [1] and 10:1 [4]. However, no sex differences have been found in the severity or location of root resorption [1].

Root resorption of the maxillary incisors is often difficult to identify on intra-oral radiographs, mainly due to the overlapping of the incisors by the ectopic canine. Cone-beam computed tomography (CT) is superior to conventional X-ray methods for the assessment of incisor root resorption associated with ectopically positioned maxillary canines, as it eliminates the blurring problem of conventional tomography and increases the perceptibility of root resorption substantially [5]. It was found that 50% more resorptions are detected with CT compared with conventional radiographic methods [6,7].

Early detection and assessment of the extent of resorption is, therefore, of fundamental importance if preventive and early corrective measures are to be taken in order to reduce later complications and to prevent the resorption from getting worse.

Interventional orthodontic treatment consists of a brief period of orthodontic therapy or the removal of teeth (deciduous and/or permanent), with the attempt to eliminate tooth impaction. In this case report we describe the use of a fixed palatal appliance with a torquing spring to move the root of lateral incisors away from the erupting canines.

## Case presentation

### Diagnosis and treatment plan

**Figure 1:**
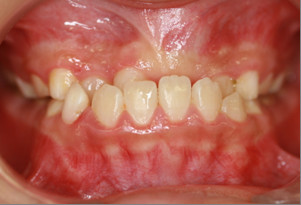
**Intra-oral frontal view showing the mixed dentition with anterior reverse overjet**.

**Figure 2:**
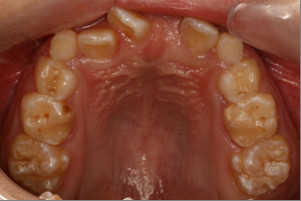
**Intra-oral upper occlusal view showing the severe crowding**.

A 10-year-old Chinese boy was referred to our Orthodontic Department at Hong Kong University with the chief complaint of delayed eruption of the upper left central incisor. The child was in good health and had no relevant medical history or previous dental trauma. Intra-oral examination showed that he had a mixed dentition, reverse anterior overjet with complete deep bite and clinically missing upper left central incisor (Figures 1 and 2).

**Figure 3:**
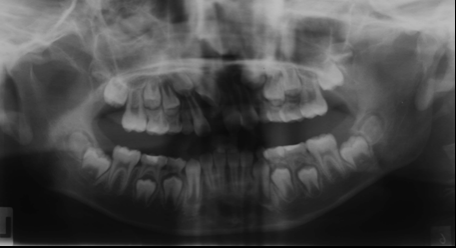
**Panoramic radiograph showing the erupting canines situated in a mesial position and close to the apices of the lateral incisors**.

Radiography showed all the permanent teeth were present and the possibility of root resorption of the upper lateral incisors (12, 22), caused by mesially erupting position canines could not be ruled out (Figure 3).

**Figure 4:**
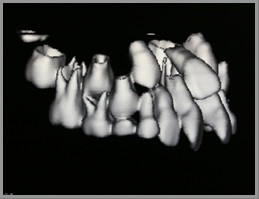
**The three-dimensional view of the cone-beam computed tomography (CT) scan showing the position of the right erupting canine**. The upper right canine was situated in close proximity to the upper right lateral incisor.

**Figure 5:**
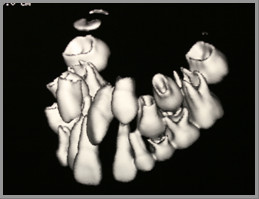
**The three-dimensional view of cone-beam computed tomography (CT) scan showing the position of the left erupting canine**. The upper left canine was hitting the root of the upper left lateral incisor.

**Figure 6:**
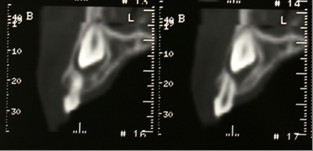
**Sequential transaxial views showing that the cusp tip of the upper left canine was located apically to the root apex of the upper left lateral incisor**. Note the resorption of the root.

A cone-beam CT scan was taken to assess the extent of resorption, if any, and to aid in the creation of a suitable treatment plan. The three-dimensional view from the CT scan (Figures 4 and 5) revealed that the crown of the upper right canine was in close proximity to the upper right lateral incisors. The crown tip of the upper left canine was touching the root of the upper left lateral incisors, causing some root resorption, which was confirmed in the sequential transaxial views of the CT scan (Figure 6).

**Figure 7:**
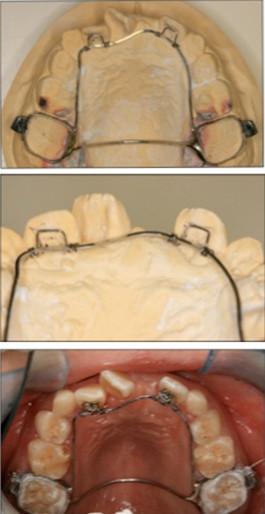
**Upper fixed palatal appliance with torquing spring used with our patient**. Top: the appliance was constructed from two molar band connected by palatal arch. A 19 × 25 stainless steel (ss) wire was extended from the molar bands to the palatal side of the upper lateral incisors. Middle: the palatal root torque was delivered to the lateral incisors by activation of the torquing spring. Bottom: tip edge brackets were bonded to the palatal surface of the upper lateral incisors and were ligated to the ss wire by using a steel ligature.

The treatment objective was to prevent and eliminate any root resorption of the lateral incisors that might be caused by the erupting canines. The treatment plan was extraction of the upper primary first molars and palatal root torque of the lateral incisors by using a specially designed upper fixed lingual appliance (Figure 7).

### Treatment progress and results

Because our patient had a reverse overjet and 100% complete overbite, the conventional labial bracket system could not be used. Therefore, a specially designed upper fixed appliance with a torquing spring was constructed to apply torque to the upper lateral incisors from the palatal side. Tip-edge brackets were bonded to the palatal surface of the upper lateral incisors for tying the palatal arch-wire into the teeth.

### Description of the appliance

A 19 × 25 stainless steel (ss) wire was soldered to the fixed palatal arch and extended anteriorly to the palatal surface of the lateral incisors. Two torquing springs were attached to the wire to rest on the palatal surface of the lateral incisors.

The appliance was cemented onto the upper first molars, and fitted anteriorly into the bracket slot and secured in place by using ligature ties through the vertical slot of the bracket. Prior to cementation the torquing spring was activated to apply a palatal root torque to the upper lateral incisors.

**Figure 8:**
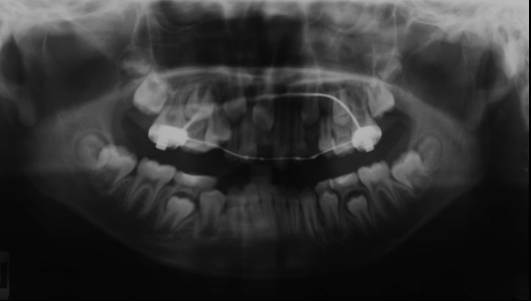
**Post-treatment panoramic radiograph showing the upper lateral incisors in a more upright position and the crown tip of the upper canine moved away from the apices of the lateral incisors**.

**Figure 9:**
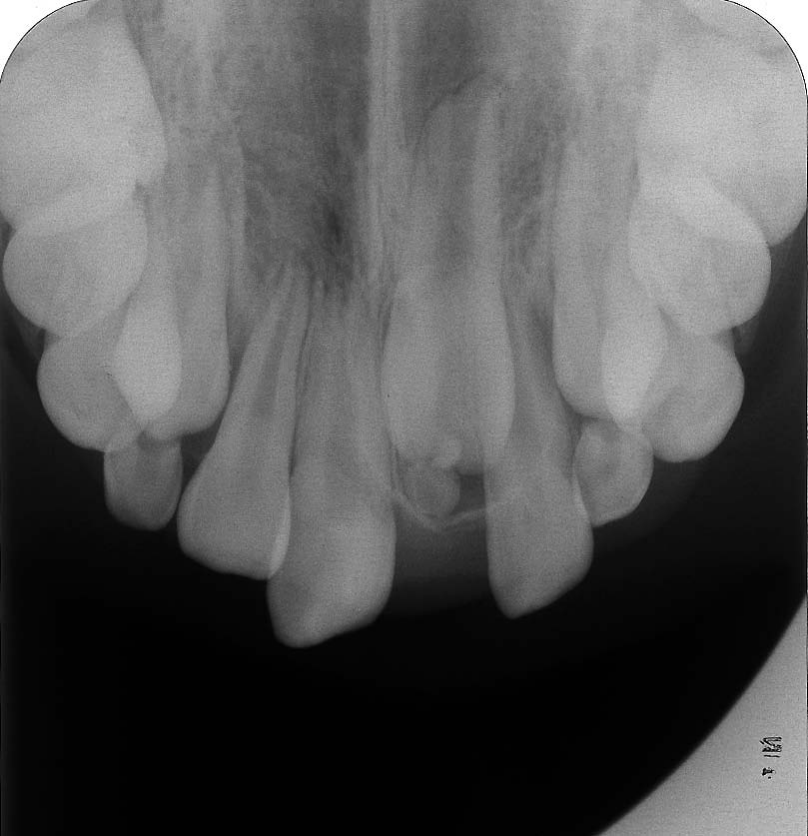
**Subsequent radiograph showing that both canines erupted further down from the roots of the lateral incisors, with no signs of root resorption**.

**Figure 10:**
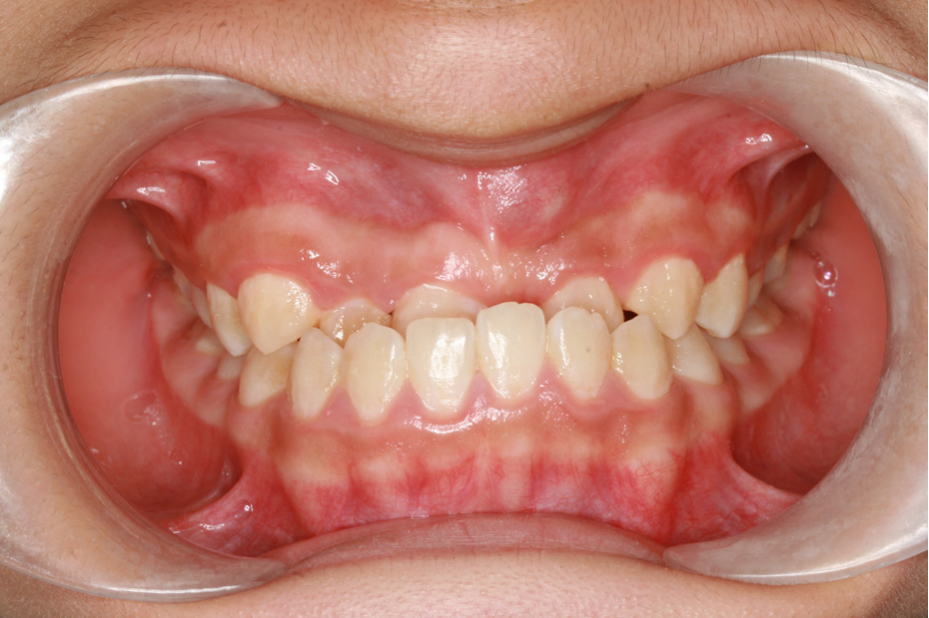
**Later photograph showing the successful eruption of both canines**.

Then, three months after the treatment, radiography showed that the lateral incisors were in a more upright position and the upper canines were erupting in a more favorable distal direction away from the root of lateral incisors, and no further resorption was evident (Figure 8). Our patient was further reviewed and a subsequent radiograph (Figure 9) showed that the both canines had erupted further down from the roots of the lateral incisors, and there were no signs of root resorption. Later photographs (Figure 10) show the successful eruption of both canines.

## Discussion

In this case report, a fixed palatal appliance was used to prevent and eliminate root resorption of the upper lateral incisors caused by the erupting canines. Resorption of adjacent teeth during the eruption of maxillary canines is rare in children. However, if it happens it can require comprehensive orthodontic treatment including extractions. Early detection and assessment of the extent of resorption is very important for treatment modality, and may decrease the extent of later complications [8].

In our patient, the main objective was to prevent and minimize root resorption of the lateral incisors due to the erupting maxillary canines. The interceptive treatment objective was achieved by applying a palatal torque to the root of the lateral incisors away from the canines and by extraction of the primary first molars, which provided more room for the canines to erupt in a more distal direction away from the roots of the lateral incisors.

Because of the excellent tissue contrast and precise three-dimensional images achievable, the use of CT scans for localization of the impactions and for evaluation of resorption is advocated by many authors. In our patient's case, a CT scan provided us with an accurate evaluation of the position of the erupting canines in relation to the roots of the lateral incisors, and as we found resorption had already started in the upper left lateral incisor and could start any time in the upper right lateral incisor, we decided to intervene immediately to save both teeth. However, the radiation dose of a CT scan is approximately 10 times to that of an orthopantomogram [9]. Therefore, once we ascertained that the tip of the canine had passed away from the apex of the lateral incisor using conventional radiography, the chance of root resorption of the lateral incisor was small and another cone-beam CT scan to reconfirm the situation was not justified.

One may question why extraction of the primary canines was not selected to provide the increased space needed for the eruption of the permanent canines in this case. Our explanation is that the plan was to allow the upper canines to erupt distal to the root apices of the lateral incisors. The primary canines were intentionally not extracted so that the lateral incisors would not drift distally, which would in turn have prevented the canines from erupting distally.

The reverse overjet was not corrected at this stage for two reasons. The first reason is our patient has a significantly increased mandibular length, therefore the chances of relapse to reverse overjet would be great. The second reason is the root of right central incisor is in contact with the crown of the unerupted left central incisor, as shown in the cone-beam CT. To move the right central incisor may have increased the risk of root resorption. For the upper left central incisor, a later radiograph showed the appearance of odontome-like structures that were not apparent in the original cone-beam CT. The odontomes were subsequently removed surgically and a bracket was bonded onto the upper left central incisor for orthodontic extrusion.

Evaluation of our patient after treatment showed an acceptable outcome. This case illustrates the importance of monitoring canine eruption and taking immediate action if necessary. Further monitoring on the erupting teeth will be carried out.

## Conclusions

Close monitoring of erupting canines is very important and appropriate intervention is critical to avoid or minimize root resorption. Early intervention can spare the patient time, expense, more complex treatment and injury to otherwise healthy teeth.

## Consent

Written informed consent was obtained from the parent of the patient for publication of this case report and accompanying images. A copy of the written consent is available for review by the Editor-in-Chief of this journal.

## Competing interests

The authors declare that they have no competing interests.

## Authors' contributions

RWKW designed the fixed appliance, supervised the treatment of our patient and revised the manuscript. BKA conducted the treatment and was a major contributor in writing the manuscript. All authors read and approved the final manuscript.
